# Interplay between dendritic non-linearities and STDP

**DOI:** 10.1186/1471-2202-12-S1-P338

**Published:** 2011-07-18

**Authors:** Matthieu Gilson, Tomoki Fukai, Taro Toyoizumi

**Affiliations:** 1 Lab for Neural Circuit Theory, RIKEN Brain Research Institute, Wako-shi, Saitama,351-0198, Japan; 2 RIKEN Brain Research Institute, Wako-shi, Saitama,351-0198, Japan

## 

Recent results about dendritic computation of responses to presynaptic stimulations have raised a lot of interest. In particular, the integration of postsynaptic potentials (PSPs) exhibits non-linearities depending on their location on dendrites, even before it reaches the soma [1]. This implies important implications for spike processing at the scale of the whole neuron, when stimulated by many input spike trains via distributed synapses. Here we examine a specific aspect of dendritic non-linearities related to spike-timing-dependent plasticity (STDP). Under STDP, synapses are strengthened or weakened depending on the relative timing of pre- and postsynaptic spikes at the scale of milliseconds. The resulting learning dynamics can dramatically shape the distribution of synaptic weights, which in turn modifies the neuronal response to its inputs [2]. We investigate the interplay between dendritic non-linearities, weight distributions and STDP-based learning, in order to evaluate whether their combined effects can be useful in terms of spiking computation.

We develop a mathematical analysis using the Poisson neuron model (spiking neuron) and a phenomenological model of STDP that describes the weight update using a learning window function [3]. The synaptic competition depends on properties of the STDP learning window, non-linear PSP integration, and stimulating inputs (in particular, the input spike-time correlations). As a first step, we constrain the present study to a configuration where pools of input spike trains with (narrow) temporal correlations excite certain groups of synapses. In addition, we assume linearity in the summation of PSPs for proximal (basal) synapses, whereas PSPs on distal (apical) synapses experience a supralinear summation when they belong to the same branch [1].

Using our mathematical framework, we show how distinct STDP rules applied to proximal and distal synapses can “match” their different PSP properties. Because of the corresponding supralinearity, a small increase of the weights for distal synapses will be felt stronger at the soma, as compared to proximal synapses. In this sense, a Gaussian-like distribution for the former is equivalent to a long-tail weight distribution for the latter. For linear PSP summation, such distributions can be produced by multiplicative STDP [2] and a new weight-dependent STDP model that we proposed recently (submitted work), respectively. Here we discuss how these distributions are affected by the dendritic non-linearities. Then we investigate how some synapses are potentiated at the expense of others for various input configurations, in particular, the possibility of jointly selecting correlated inputs even though they excite different dendritic sites.

A previous study [4] used additive (weight-independent) STDP with a realistic neuron model in order to examine the competition between basal and apical synapses. It was found in this configuration that, in most cases, either basal or apical synapses take over, and a balance between them is difficult to obtain. Our results suggest that non-additive STDP models can partly solve this problem by adjusting their weight dependence to the dendritic non-linearity, and realize a “fair” synaptic competition. This means that the emerging connectivity can reflect the stimulating input properties despite the constraint of being located on different dendritic branches. A future step consists in incorporating more physiological details in our model (e.g., compartmental neuron model as in [4]) to verify that our results still hold. Eventually, we aim to understand how the diversity of STDP and PSP properties observed in the biology contributes to the computational power of neurons in terms of spike processing.

## References

[B1] London M Häusser M Dendritic computation Ann Rev Neurosci 2005 28 503 532 10.1146/annurev.neuro.28.061604.135703 16033324

[B2] van Rossum MCW Bi GQ Turrigiano GG Stable Hebbian learning from spike timing-dependent plasticity J Neurosci 2000 20 8812 8821 11102489 10.1523/JNEUROSCI.20-23-08812.2000 PMC6773092

[B3] Gilson M Burkitt AN van Hemmen JL STDP in recurrent neuronal networks Front Comput Neurosci 2010 4 23 20890448 10.3389/fncom.2010.00023 PMC2947928

[B4] Ilan LB Gidon A Segev L Inter-regional synaptic competition in neurons with multiple STDP-inducing signals J Neurophysiol 2010 online-first version (PMID:21123659) 21123659 10.1152/jn.00612.2010

